# The out-of-pocket cost of breast cancer care at a public tertiary care hospital in Nigeria: an exploratory analysis

**DOI:** 10.11604/pamj.2022.41.272.24610

**Published:** 2022-04-05

**Authors:** Gregory Christopher Knapp, Funmilola Olanike Wuraola, Olalekan Olasehinde, Anya Romanoff, Peter Thomas Kingham, Olusegun Isaac Alatise

**Affiliations:** 1 Department of Surgery, Division of General Surgery, Dalhousie University, Halifax, Nova Scotia, Canada,; 2 Department of Surgery, College of Health Sciences, Obafemi Awolowo University (OAU), Ile-Ife, Nigeria,; 3 Memorial Sloan Kettering Cancer Center, New York, New York, United States of America

**Keywords:** Out-of-pocket cost, financial toxicity, breast cancer, Nigeria

## Abstract

**Introduction:**

in Nigeria, the incidence of breast cancer has increased by over 80% in the last four decades. This study quantifies the out-of-pocket (OOP) cost of breast cancer management and the associated rate of catastrophic healthcare expenditure (CHE) at a public tertiary care facility in Ile-Ife, Nigeria.

**Methods:**

patients treated between December 2017 - August 2018 were identified from a prospective breast cancer database. A questionnaire was developed to capture the total cost of care, including direct and indirect expenses. Three commonly used thresholds for a CHE were used in this analysis. The cost of radiotherapy and targeted therapy were captured separately.

**Results:**

data was collected from 22 eligible patients. Sixty-eight percent had no form of health insurance. The mean cost of diagnosis and treatment was $2,049 (SD $1,854). At a threshold of 10% and 25% of annual income, 95% and 86% of households experienced a CHE. Based on a household´s capacity-to-pay, 90% experienced a CHE. The mean cost of radiotherapy was $462 (SD $223) and the mean cost of trastuzumab was $6,568 (SD $2,766). Cost precluded surgery in 14% of patients with resectable disease. As a result of accessing treatment, 72% of households had to borrow money and 9% of households interrupted a child´s education.

**Conclusion:**

the out-of-pocket cost of breast cancer care in Nigeria is significant. This results in a CHE for 68-95% of households, which has significant health and economic sequelae. Greater financial protection is essential as the burden of breast cancer increases in Nigeria.

## Introduction

The incidence of breast cancer is increasing across sub-Saharan Africa [1]. In Nigeria, the number of new cases nearly doubled between 1960 - 2000 [1]. It is now the most common cause of cancer-related death with an age-standardized incidence over 20 per 100,000 [2,3]. As the burden of cancer increases, the manner in which the healthcare system finances the delivery of care is increasingly relevant to policy makers and clinicians [4,5]. Without financial protection, a diagnosis of breast cancer can initiate a poverty-illness cycle characterized by inadequate treatment, poor outcomes, and deeper impoverishment [6,7]. Late presentation and advanced disease, which are common in Nigeria, exacerbate this cycle, as costly treatment is less likely to improve outcomes [8,9]. Although Nigeria has a large public healthcare system, government spending (i.e. 0.5% of gross domestic product) only accounts for 13% of total healthcare expenditures [10,11]. The National Health Insurance Scheme (NHIS) provides health insurance through involuntary (e.g. formal sector health insurance program) and voluntary (e.g. for the self-employed or retired) enrolment [12]. However, less than 10% of the population has some form of health insurance (i.e. public or private) [13-15]. The majority of healthcare spending is out-of-pocket (OOP), which is associated with a high rate of catastrophic healthcare expenditure (CHE) [13,16-21]. There is a paucity of data on the unique costs of cancer care and the implications for patients and families in Nigeria. This poses a barrier to the creation of effective health policy and local treatment guidelines. This study presents an analysis of the direct and indirect costs of breast cancer diagnosis and management at a public university teaching hospital in South West, Nigeria. The degree of financial protection from the cost of illness is examined by quantifying the rate of catastrophic spending and the non-financial implications of accessing care.

## Methods

**Study population:** six hundred and thirty-five patients were identified from a prospectively maintained breast cancer database between May 2009 - January 2019. This database is maintained by the African Research Group for Oncology (ARGO) and captures data on sociodemographics, clinical presentation, histopathology, treatment (i.e. surgery, chemotherapy, radiotherapy), and long-term outcomes. The staging system used in this database reflects the American Joint Committee on Cancer 7^th^ edition. All patients who underwent treatment between December 2017 - August 2018 were contacted for enrollment and asked to provide information regarding the cost of their treatment. This enrollment window was chosen to ensure the full cost of treatment was captured, including the cost of adjuvant chemotherapy, while minimizing the impact of recall bias. To capture the OOP cost associated with targeted therapy for cancers overexpressing human epidermal growth factor-2 (HER-2) and radiotherapy, the entire database (May 2009 - January 2019) was queried. Patients receiving either therapy were contacted regarding the cost of this aspect of care. This information was gathered with a separate interviewer-administered questionnaire. Oral consent was obtained from all patients and/or attendants who agreed to participate. The consent process and questionnaire were administered in either English or Yoruba.

**Quantifying out-of-pocket costs and catastrophic healthcare expenditure:** this study defined any payment for services directly (e.g. cost of medicine) or indirectly (e.g. cost of travel) associated with accessing care, without reimbursement, as an OOP cost. A CHE was broadly defined as spending that reduced household consumption of basic goods [22-24]. We used three commonly employed thresholds for calculating a CHE, including 10% and 25% of total household income [19,25,26] and ≥ 40% of a household´s capacity-to-pay. A household´s capacity-to-pay was defined as annual household income net the cost of annual subsistence needs, such as food [23,24]. In many low- and middle-income countries (LMICs), where income may not be a reliable indicator of purchasing power, capacity-to-pay is a more robust measure of effective income [23]. These three thresholds (i.e. 10% and 25% of income, 40% capacity-to-pay) were used to calculate the rate of CHE once total OOP spending was quantified for each household. Annual household income and capacity-to-pay data were self-reported using a detailed interviewer-administered questionnaire. Average annual capacity-to-pay for the hospital´s catchment area was also derived from the most recent General Household Survey [27]. Personal and household income were converted from the local currency (Naira) to dollars (USD$) using the Central Bank of Nigeria´s conversion rate on May 2, 2019 (N=305.95).

**Questionnaire:** a novel questionnaire was developed to capture a detailed inventory of breast cancer care related costs, household income and capacity-to-pay. Study participants were identified from the prospective database and enrolled during routine follow-up. Consent for the study was obtained and the questionnaire was administered in the outpatient clinic by research personnel. Patients were asked to estimate their household monthly income from all sources. This was followed by a detailed assessment of monthly household expenditures to determine each household´s discretionary, non-subsistence income (capacity-to-pay). Finally, to benchmark the study population against regional and national norms, components of the Oxford Multidisciplinary Poverty Index (MPI) were elicited for each patient [28]. To determine the OOP costs for each patient, the care continuum was subdivided into three phases: pre-op evaluation, including neoadjuvant chemotherapy; inpatient care, including surgery; and post-op care, including adjuvant systemic therapy and radiation. The questionnaire was based on a similar design by Anderson *et al*. (2017) and was intended to capture all direct and indirect (e.g. lost income, lodging) OOP costs associated with each phase of care [26]. To reduce recall bias, patients and attendants were asked to refer to their hospital assessment fee ledger and any receipts relevant to the cost of their treatment when completing the questionnaire. Furthermore, the cost of specific procedures (e.g. modified radical mastectomy) and medications (e.g. chemotherapy) were verified with the hospital accounting department or local distributor to minimize recall bias. The indirect cost of care included the cost of travel, supplemental lodging and food and an estimate of lost-income. The cost of travel was calculated for the patient and any accompanying family members per visit to the hospital and multiplied by the trip frequency. If patients or family were from out of town, the cost of supplemental lodging and food was also elicited. The patient was also asked to estimate the total amount of income forfeited or lost as a result of illness. In addition to capturing the total OOP cost of care, the impact of cost on treatment course and duration was explored. The impact of OOP costs on other aspects of daily living and household wellbeing were also elicited, such as job loss resulting from illness, the need to sell land or possessions, the need to remove a child from school, and the requirement to borrow or take on debt to pay hospital bills.

**Data analysis:** the primary objective of the analysis was to quantify the OOP cost and percentage CHE resulting from breast cancer care for a defined population in Nigeria. The secondary objective was to examine whether there was a statistically significant difference in the percentage of CHE between those with and those without health insurance. Descriptive statistics were used to present numerical and categorical sociodemographic and clinicopathological variables. For each patient, the total OOP costs for all three phases of care (i.e. pre-op, peri-op and post-op) was calculated from the questionnaire data provided. In a similar manner, total annual household income and capacity-to-pay was estimated for each participant. For each participant, the data was analyzed to determine if total OOP costs exceeded either 10% or 25% of self-reported annual household income or 40% of annual capacity-to-pay. If total OOP costs exceeded one of the three expenditure thresholds (i.e. 10% or 25% annual income, 40% capacity to pay) a patient was deemed to have experienced a CHE. The mean OOP cost for breast cancer care was also calculated for the study population. The mean annual household income and capacity-to-pay were also calculated for the study population. A one-sample Student´s t-test was used to compare the mean capacity-to-pay of the study cohort with the mean capacity-to-pay for the geopolitical zone owing to the unknown variance in the population mean (i.e. mean capacity-to-pay for the geopolitical zone). A two-sample Z-test was used to compare the rate of CHE between two populations; those with and those without health insurance. Statistical analysis was performed using Stata 14 (StataCorp. 2015. *Stata Statistical Software: Release 14*. College Station, TX: StataCorp LP). A significant result was defined as a p<0.05.

**Ethical approval:** the collection and maintenance of the prospective database has been approved by the Independent Research Board at Obafemi Awolowo University (OAU). A separate research protocol for this study, including the administration of the questionnaire, was granted ethical clearance by OAU. Verbal and written consent was obtained from all participants. Data was collected in hardcopy and transferred to a secure electronic database without identifying information.

## Results

**Figure 1:**
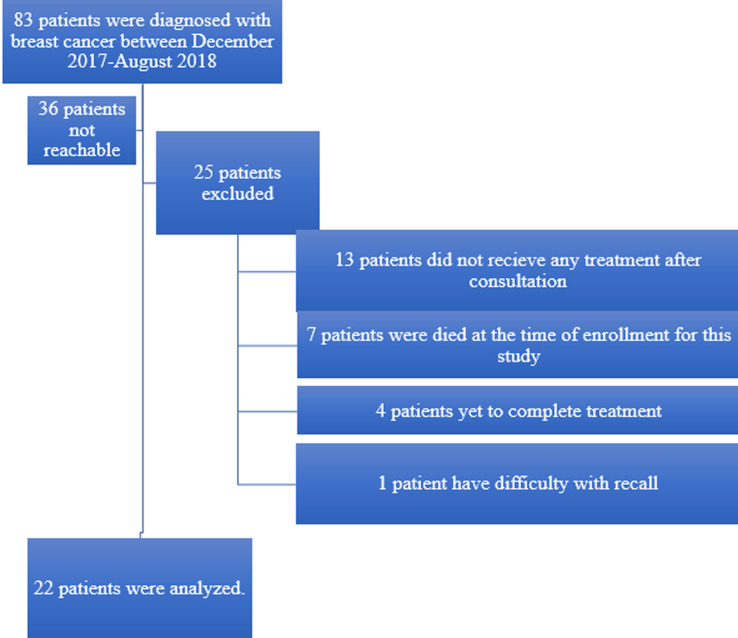
PRISMA diagram outlining patient eligibility and exclusion

Eighty-three patients were diagnosed with breast cancer and entered into the ARGO prospective database between December 2017 - August 2018. All 83 patients were contacted for enrolment in the study of whom 36 were not reachable (e.g. outdated contact information) or declined to participate. Of the 47 remaining patients 25 were ultimately excluded, leaving a total of 22 patients successfully recruited with complete questionnaire data. Of the 25 patients who were excluded, 13 did not receive active treatment after initial consultation, seven were deceased at the time of enrollment, four had not yet completed treatment, and one individual could not adequately recall all components of care. Patient enrolment is outlined in Figure 1. The sociodemographic and clinical characteristics of those included in the analysis are presented in Table 1. The mean age of the study participant was 50.6 years and the mean size of the household was five. Nineteen of 22 (86%) completed at least secondary education and 7/22 (32%) were enrolled in the NHIS. One patient had private health insurance to supplement their NHIS coverage. The majority of patients had locally advanced disease (64% Stage III and 14% Stage IV). Ninety-five percent (21/22) received either neoadjuvant or adjuvant chemotherapy. The most common agents were epirubicin, cyclophosphamide, fluorouracil, and docetaxel. Patients received an average of 4.3 cycles of neoadjuvant and 3.8 cycles of adjuvant chemotherapy. None of the 22 patients received radiotherapy as part of their management.

**Table 1:** sociodemographic and clinical characteristics of study cohort

Variable	Mean	Median
Age	50.63	51.5
Size of household	5.36	5.5
	**N**	**%**
Level of education		
- ≤ primary	3	13.64
- secondary	10	45.45
- > secondary	9	40.91
Stage of disease at presentation		
- I	0	0
- II	5	22.72
- III	14	63.64
- IV	3	13.66
Immunohistochemistry performed	4	18.18
Received chemotherapy (neo and/or adjuvant)	21	95.45
Received radiotherapy	0	0

Data source African Research Group on oncology breast cancer database 2017-2018, Ile-Ife, Nigeria

**Household income and capacity-to-pay:** the study population´s mean self-reported annual household income was $3,645 (SD $5,828) and mean capacity-to-pay was $2,328 (SD $2,660, (Table 2). The study population was more affluent then average household in South West Nigeria ($2,328 vs. $877 SW, p=0.024). The Oxford MPI was also used to benchmark the socioeconomic status of the study population. Compared to the regional average, fewer patients were poor or vulnerable to poverty (9% vs. 23% in Nigeria).

**Table 2:** mean purchasing power of study cohort

Purchasing power	Mean (SD)
Annual household income	$3,645* ($5,828)
Annual capacity-to-pay**	$2,328 ($2,660)

* Local currency (Naira) was converted to US dollars ($) using the Nigerian Central Bank's May 1, 2019 conversion of USD=N305.95 ** Capacity-to-pay was defined as annual household income net the cost of annual subsistence needs, such as food

**Out-of-pocket and catastrophic health expenditure:** the mean OOP cost of breast cancer diagnosis and management was $2,049 (SD $1,854) for the study population. Direct costs, such as the cost of diagnostic imaging, medication and surgery, accounted for 61% of the total OOP costs associated with care. Indirect costs, including the cost of travel, supplementary lodging, and lost income, accounted for the remaining 39% of the reported mean OOP costs. For the study population, the mean direct costs of surgery were $692 (SD $336). A breakdown of the OOP costs associated with each component of care for the study population is outlined in Table 3. The rate of CHE was calculated at three commonly used thresholds (i.e. 10%, 25% of annual income and 40% capacity-to-pay). Ninety-five percent of patients accrued OOP costs that exceeded 10% of annual household income, while 86% of patients spent over 25% of annual household income. Thus, we found that between 86-95% of patients experiences a CHE based on the definition of 10% and 25% of annual income. At a threshold of 40% of a household´s capacity-to-pay, 90% experienced a CHE as outlined in Table 4. The direct cost of surgical management alone (i.e. pre-operative imaging/diagnosis, surgery, post-operative care) was associated with a CHE in 78%, 67%, and 53% of individuals at all three thresholds (i.e. 10% / 25% annual income, 40% capacity-to-pay), respectively. The mean cost of care for individuals with health insurance was $1,602 (SD $1,945). Using the definition of a CHE as an OOP expenditure that exceeds 40% capacity-to-pay, there was no difference in the rate of CHE between those with insurance and those without (80% vs. 73%, p=0.765, Table 4).

**Table 3:** direct and indirect out-of-pocket costs associated with breast cancer management

Component of care	Mean cost
Direct expenditures	
Consultation, laboratory tests, and diagnostic imaging	$141.72
Neo adjuvant chemotherapy	$304.54
Surgery + immunohistochemistry	$524.35
Adjuvant chemotherapy	$376.38
Post-operative care	$138.66
**Indirect expenditures**	
Travel	$65.65
Lodging	$10.42
Lost income	$840.90

**Table 4:** rate of catastrophic healthcare expenditure for breast cancer care and among patients with National Health Insurance Scheme (NHIS) coverage versus those without

	Threshold for a catastrophic healthcare expenditure (%)		
	10% annual income	25% annual income	40% capacity-to-pay**
**Total cost of care**	95.2	85.7	90.0
**Total cost without lost income**	95.2	81.0	75.0
**Surgery alone**	77.8	66.8	52.9
Rate of catastrophic healthcare expenditure	Patients with NHIS n=5	Patients without NHIS n=15	p-value
40% capacity-to-pay***	4(80)	11(73.3)	0.765#

* Calculated as the percentage of the study population with an OOP cost that exceeded 10% or 25% of self-reported annual household income ** Capacity-to-pay was defined as annual household income net the cost of annual subsistence needs, such as food *** Capacity-to-pay was defined as annual household income net the cost of annual subsistence needs, such as food # P-value calculated using a two-sample Z-test

None of the subjects (0/22) included in the above presented cost analysis (i.e. diagnosed between December 2017 - August 2018) received radiotherapy. Thus, the entire prospective breast cancer database was queried to generate an estimate of this particular aspect of care. Between 2009 - 2019, 50/635 patients (8%) received radiotherapy. One of these individuals was deceased, one could not reliably recall treatment related expenses, and 33 were unreachable. In total, 15/50 eligible patients were able to provide data on the OOP cost associated with the receipt of radiotherapy as a component of their breast cancer management. The mean cost of radiotherapy treatment, including direct and indirect expenses was $462 (SD $223). During the same period (2009-2019), 30/635 patients were HER-2 positive, of whom 13% (4/30) received trastuzumab. Only two of the four patients who received targeted therapy consented to participate; of the two who were not included, one declined to participate and the other was deceased. The mean OOP cost of trastuzumab, including direct and indirect expenses, was $6,568 (SD $2,766) for 7.5 doses.

**Impact of OOP expenditures:** the cost of care had an impact on treatment course or duration in 27% (6/22) of patients, including three (14%) individuals who did not undergo surgery for potentially resectable disease. Twenty-three percent (5/22) of individuals had to sell land or possessions, while nine percent (2/22) had to withdraw children from school to pay for treatment. To cover the cost of care, 72% (16/22) of households had to borrow money. As a result of seeking care for breast cancer, 18% (4/22) of patients indicated that they or a family member lost a job. These sequeala of treatment are presented in Table 5.

**Table 5:** impact of out-of-pocket costs for breast cancer diagnosis and management on patients and families

Impact	Proportion affected (%)
Borrowed money	72.28
Declined recommended treatment	27.27
Sold land and/or possessions	22.73
Lost job as a result of treatment	18.18
Withdrew children from school	9.09

## Discussion

An important metric of health system performance is the degree to which it systematically protects individuals from financial shock or impoverishment due to ill health [29]. Without a robust system of health insurance in Nigeria, most individuals pay OOP for services at the point of use [11,14,30]. This results in a high rate of CHE for elective and non-elective care across the healthcare system [18-20,31]. In this exploratory analysis, the mean OOP cost of breast cancer diagnosis and management was $2,049. This is in a similar range with previously published estimates from other LMICs, including Vietnam ($975) and Malaysia ($2,595 for colorectal cancer) [32,33]. However, significant methodological differences, including omission of direct non-medical expenses (e.g. travel cost) and indirect expenses (e.g. lost income), make a meaningful comparison difficult. Despite a wealthier than average cohort, between 86-95% of households in our study experienced financial catastrophe. This is higher than in middle-income countries such as Iran (60.9%) and China (58.1%) but similar to rates of CHE for other services within the Nigerian healthcare system [34,35]. To our knowledge, this is the first estimate of the OOP cost and rate of CHE for breast cancer care in West Africa. The impact of inadequate financial risk protection was significant. Most notably, 14% of women did not undergo potentially curative surgery and more than one in four (27%) did not receive the recommended treatment course or duration due to cost. Moreover, almost 10% of families had to interrupt a child´s education to meet their financial obligations. These metrics of financial toxicity characterize the poverty-illness cycle that pervades the healthcare systems of many LMICs and has significant implications for broader economic growth and development.

None of the patients in our study had post-operative radiotherapy, despite two-thirds presenting with locally advanced disease. A seperate analysis of the cost associated with radiotherapy suggests that both physical access and affordability are major barriers to utilization. Only 33% of patients received radiotherapy at the nearest comprehensive cancer centre (i.e. University College Hospital, Ibadan, Nigeria). The cost of HER-2 targeted therapy was even more selective. Only 13% of women with HER-2 positive disease, between May 2009 - January 2019, received targeted therapy, which on average cost $6,568 for a short-course regime. These findings suggest that women of similar need do not appear to be receiving similar care, secondary to cost and inadequate financial protection. Numerous patients in our study had coverage through the NHIS (32%), which reflects the study´s enrichment for patients with higher education and public-sector employment. Patients realized a reduction in their OOP costs with pre-paid insurance. Although this did not result in a significantly lower rate of CHE, this needs to be examined in a larger prospective study. The impact of the NHIS within the cancer care system is limited by its breadth (i.e. covered services) and depth (i.e. proportion of the population) of coverage [36]. For instance, the cost of radiotherapy is not covered by the NHIS. Targeted therapy, such as trastuzumab, is also not included in the benefit package. The first cost-effectiveness analysis of HER-2 targeted therapy in sub-Saharan Africa by Gershon *et al*. (2019) suggests that trastuzumab is not cost-effective in Nigeria [37]. According to the Breast Health Global Initiative, trastuzumab is only considered appropriate in enhanced and maximal resource settings, which further justifies its exclusion from NHIS coverage [38]. Commonly used chemotherapeutics, such as epirubicin and docetaxel, are covered by the NHIS with a 10% co-payment. However, chemotherapy is often purchased from private distributors, not registered with a designated NHIS payer (i.e. local health maintenance organization), and thus goes uncovered. In our small, exploratory study, the mean cost of chemotherapy was 18% of the total cost of care and none of the patients with NHIS coverage received reimbursement for the cost of chemotherapy.

There are numerous limitations of this study, including the small sample size and single institution, retrospective design. Data was collected from patients treated at a single teritary care center in South West Nigeria and may not represent the costs of care in other regions of the country. The data should not be extrapolated to other countries in West Africa. Although only 27% (22/83) of eligible patients were included in the analysis, the study population had a similar sociodemographic profile to those excluded. The stage at presentation was also similar between those included (63% stage III) and those excluded from the analysis (64% Stage III). Given the relative affluence of those included, compared to the mean household capacity-to-pay for the region, we suspect that our analysis reflects a conservative estimate of the degree of financial toxicity experienced by breast cancer patients in the rural South West of Nigeria. The cost of trastuzumab was only captured for two individuals and is likely not representative of the current cost of treatment in Nigeria. Similarly, the historical date range used for enrolment of patients receiving radiotherapy may not reflect the current state of availability or proximity. We attempted to minimize the impact of recall bias by limiting the date range in the primary analysis to the most recent cohort of patients who had completed treatment. Self-reported expenses were referenced against known fixed costs provided by the hospital´s accounting department and personal communication with local suppliers. Self-reported income was also not validated. Thus, our aggregated capacity-to-pay measurement may be a more accurate representation of purchasing power.

The growing burden of cancer in Nigeria highlights the degree to which the current system fails to provide adequate protection from the cost of illness. Expansion of prepayment and risk pooling will be essential to address these shortcomings. This may be possible through the existing NHIS; however, voluntary enrollment is unlikely to achieve the goal of universal coverage [36]. The federal government previously committed to spending 15% of its annual budget on healthcare, as a signatory of the 2001 Abuja Declaration [39]. Unfortunately, current spending remains well below this threshold [11]. The breadth and depth of NHIS coverage could be expanded with government spending on healthcare that targets 15% of the budget. Country-specific cost-effectiveness data is needed to guide the expansion of cancer care benefits. This should accompany the adoption of resource sensitive treatment guidelines for the cancers outlined in the National Cancer Control Plan [5]. As highlighted in our analysis, a more nuanced understanding of the unique barriers to effective financial protection, including reimbursement for covered services unavailable at registered facilities, will be important to address the gaps in the current system.

## Conclusion

Access to healthcare is dependent on services being physically accessible, affordable, and acceptable to the target population [40]. This study begins to address the pressing need for a more granular understanding of the cost of cancer care in Nigeria. In our small, exploratory analysis, the OOP cost for breast cancer care resulted in a rate of CHE of 86-95%. This had a negative impact on the provision of care and the material and intellectual capital of affected households. Greater financial protection is essential as the burden of breast cancer increases in Nigeria.

### What is known about this topic

- Breast cancer is the leading cause of cancer related mortality in Nigeria;
- The cost of modern, multidisciplinary cancer care is significant and without adequate financial protection can lead to catastrophic personal healthcare expenditures with long-term sequelae for the patient and household;
- Little is known about the out-of-pocket cost of breast cancer care in Nigeria.

### What this study adds

- Over 85% of patients experience a catastrophic healthcare expenditure as a result of breast cancer diagnosis and treatment at a single tertiary care facility (public) in South West Nigeria;
- One in four women in our study did not receive the recommended treatment due to the financial burden of the interventions;
- Enrollment in the Nigerian National Health Insurance Scheme (NHIS) does not result in a significant decrease in the rate of catastrophic healthcare expenditure for women with breast cancer compared to patients without NHIS coverage.
